# About classical molecular genetics, cytogenetic and molecular cytogenetic data not considered by Genome Reference Consortium and thus not included in genome browsers like UCSC, Ensembl or NCBI

**DOI:** 10.1186/s13039-021-00540-7

**Published:** 2021-03-20

**Authors:** Thomas Liehr

**Affiliations:** Jena University Hospital, Friedrich Schiller University, Institute of Human Genetics, Am Klinikum 1, 07747 Jena, Germany

**Keywords:** Genome Reference Consortium (GRC), International System for Human Cytogenetic Nomenclature (ISCN), International System for Human Cytogenomic Nomenclature (ISCN), Subbands, Satellite DNA

## Abstract

**Background:**

The Genome Reference Consortium (GRC) has according to its own statement the “mission to improve the human reference genome assembly, correcting errors and adding sequence to ensure it provides the best representation of the human genome to meet basic and clinical research needs”. Data from GRC is included in genome browsers like UCSC (University of California, Santa Cruz), Ensembl or NCBI (National Center for Biotechnology Information) and are thereby bases for scientific and diagnostically working human genetic community.

**Method:**

Here long standing knowledge deriving from classical molecular genetic, cytogenetic and molecular cytogenetic data, not being considered yet by GRC was revisited.

**Results:**

There were three major points identified: (1) GRC missed to including three chromosomal subbands, each, for 1q32.1, 2p21, 5q13.2, 6p22.3 and 6q21, which were defined by International System for Human Cytogenetic Nomenclature (ISCN) already back in 1980s; instead GRC included additional 6 subbands not ever recognized by ISCN. (2) GRC defined 34 chromosomal subbands of 0.1 to 0.9 Mb in size, while it is general agreement of cytogeneticists that it unlikely to detect chromosomal aberrations below 1–2 Mb in size by GTG-banding. And (3): still all sequences used in molecular cytogenetic routine diagnostics to detect heterochromatic and/ or pericentromeric satellite DNA sequences within the human genome are not included yet into human reference genome. For those sequences, localization and approximate sizes have been determined in the 1970s to 1990, and if included at least ~ 100 Mb of the human genome sequence could be added to the genome browsers.

**Conclusion:**

Overall, taking into account the here mentioned points and correcting and including the data will definitely provide to the still not being completely finished mapping of the human genome.

**Supplementary Information:**

The online version contains supplementary material available at 10.1186/s13039-021-00540-7.

## Background

The goal to understand ourselves as human beings and what makes us that different from all other species on the planet through centuries led to multiple lines of sciences, including philosophy, theology, history, medicine and natural sciences. The latter discipline gave birth to genetics and initiated ~ 3 decades ago the effort to sequence the entire human genome, with the hope to finally reach here an in-depth breakthrough concerning the above mentioned question.

Still, one can see the enthusiasm the ‘Human Genome Project’ (HGP) was and is accompanied by, in the statement on the corresponding internet presence as: “The HGP was one of the great feats of exploration in history. Rather than an outward exploration of the planet or the cosmos, the HGP was an inward voyage of discovery led by an international team of researchers looking to sequence and map all of the genes—together known as the genome—of members of our species, *Homo sapiens.* Beginning on October 1, 1990 and completed in April 2003, the HGP gave us the ability, for the first time, to read nature's complete genetic blueprint for building a human being” [1].

Looking at the history of human genetics, it was Gregor Mendel who suggested 1856 that in the cells there must be “coupling groups”, i.e. that what was seen by Walther Flemming in 1879 and called by Wilhelm Waldeyer in 1888 ‘chromosomes’ [2]. Even though banding cytogenetics, introduced by Lore Zech in the late 1960s provided major progress in human genetics [3], soon the developments in molecular genetic techniques were taking over the main stream of the field [4]. However, neither a pure base pair oriented view (of molecular genetics) nor an isolated chromosome-oriented view (of (molecular) cytogenetics) alone will ever be sufficient to understand our genome. Accordingly, the Genome Reference Consortium (GRC), being responsible for collecting and publishing the human genome reference sequence, aligns actual sequencing data with the chromosomal level. Recent insights from ‘second-’ and ‘third-generation-sequencing’ approaches [5] highlighted furthermore by discovery of inter- and intrachromosomal interactions, and more specifically the TADs (topologically associating domains) [6], that chromosome structure is extremely important for genome function, and if impaired for diseases [5, 6]. Most recently, insight from electron microscopy combined with cytogenetic, molecular cytogenetic and molecular genetic data led to a complete new understanding of the chromosome structure itself [7]. As stated by Joan-Ramon Daban: “Experimental evidence indicates that the chromatin filament is self-organized into a multilayer planar structure that is densely stacked in metaphase and unstacked in interphase. This chromatin organization is unexpected, but it is shown that diverse supramolecular assemblies are multilayered. The mechanical strength of planar chromatin protects the genome integrity, even when double-strand breaks are produced.” He suggests “that the chromatin filament in the loops and topologically associating domains is folded within the thin layers of the multilaminar chromosomes. It is also proposed that multilayer chromatin has two states: inactive when layers are stacked and active when layers are unstacked” [7].

In this paper it is discussed the following: in as much could GRC profit in its mission to “improve the human reference genome assembly, correcting errors and adding sequence to ensure it provides the best representation of the human genome to meet basic and clinical research needs” [8] by performing also such an integrative view on the available data of the human genome as Joan-Ramon Daban did? This question is of immense practical meaning, as GRC-data is bases for genomic browsers like UCSC (University of California, Santa Cruz) [9], Ensembl [10] or NCBI (National Center for Biotechnology Information) [11], and those are being applied as backbone for correct interpretation of (molecular) cytogenetic and molecular genetic diagnostic results. In the following, insights to be considered from banding cytogenetics, molecular genetics and molecular cytogenetics are accordingly discussed, because as recently stated by Ye and colleagues: “karyotype coding” defines the genome system information [12].

## Insights from banding cytogenetics

Banded human chromosomes confronted scientists in the 1970s with similar problems and questions like nowadays there is with sequencing data:

- How to describe and how to denominate what we see?
- How to define a worldwide valid nomenclature for what we see?
- How to give a definite and reliable nomenclature for bands or DNA-sequence positions?

**Fig. 1:**
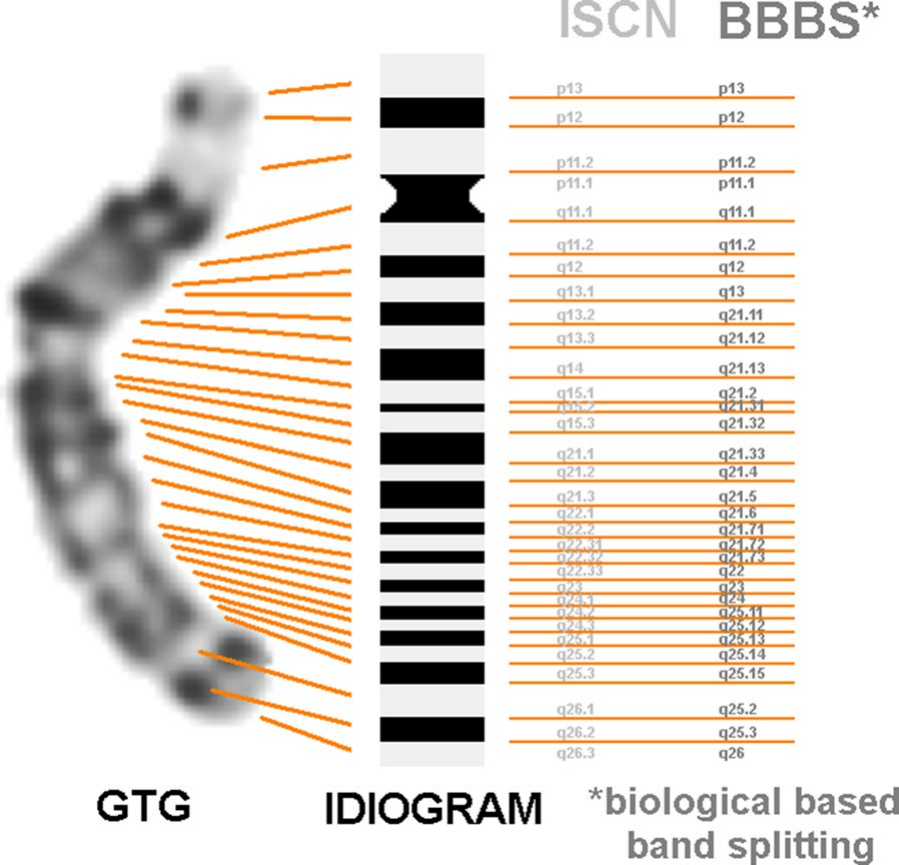
A GTG-banded human chromosome 15 at about 850 haploid band level is depicted; at its right side an idiogram is shown. The black and white bands are numbered according to an international consensus (the ISCN nomenclature—light gray caption). The ISCN based nomenclature does not describe the band splitting from shorter to larger chromosomes—this biologically based band splitting (BBBS) is different (acc. to [18] in gray caption)

Banding pattern in human chromosomes was denominated compulsorily latest in 1978 [13], and refined later on, with progress of used methods and higher banding resolution [14]. Since many editions of the International System for Human Cytogenomic Nomenclature (ISCN), banding nomenclature has not been changed, and refers to data from 1981 and 1994 [15]. This denomination of bands had just the goal to describe what is visible in a light-microscope: shorter, more condensed chromosomes show less GTG-dark and -light bands than longer, more decondensed ones (Fig. 1). However, this nomenclature is by no means reflecting biological realities, i.e. it does not describe which subbands at a higher chromosomal resolution derived from which more condensed ones, at lower resolution. This has been shown by seminal works of Uwe Claussen, who could demonstrate that GTG-light bands represent maximally decondensed chromosomal parts, and never split into further subbands. He showed this for chromosomes 6 [16] and Xp [17] using chromosomes stretching, and for all other chromosomes applying another, fluorescence in situ hybridization (FISH) based approach [18] (Fig. 1).

In as far as it might be important for GRC to consider that e.g. the maximally stretched short arm of the X-chromosome has overall 14 GTG-light and 13 GTG-dark bands, has to be seen in future. At least one could deduce from that study [17] that there might be roughly (if Xp represents ~ 2% if human genome) overall ~ 700 GTG-light and 650 GTG-dark bands in the whole human genome, which wait for their alignment with sequencing data. In the light of the description of TADs [6] and work of Joan-Ramon Daban [7] this has to be considered earlier or later. Also an important clue to learn from this work [16–18], is that in GTG-light bands, being less condensed than GTG-dark ones, there should be less DNA included than in more condensed dark ones. The GTG-dark bands, being alinged at ~ 850 band level with sequencing results in genome browsers, can still be decondensed and should thus must contain more DNA and more GTG-light and dark subbands at higher decondensation levels.

### There are lacking and newly postulated subbands in GRC

**Fig. 2:**
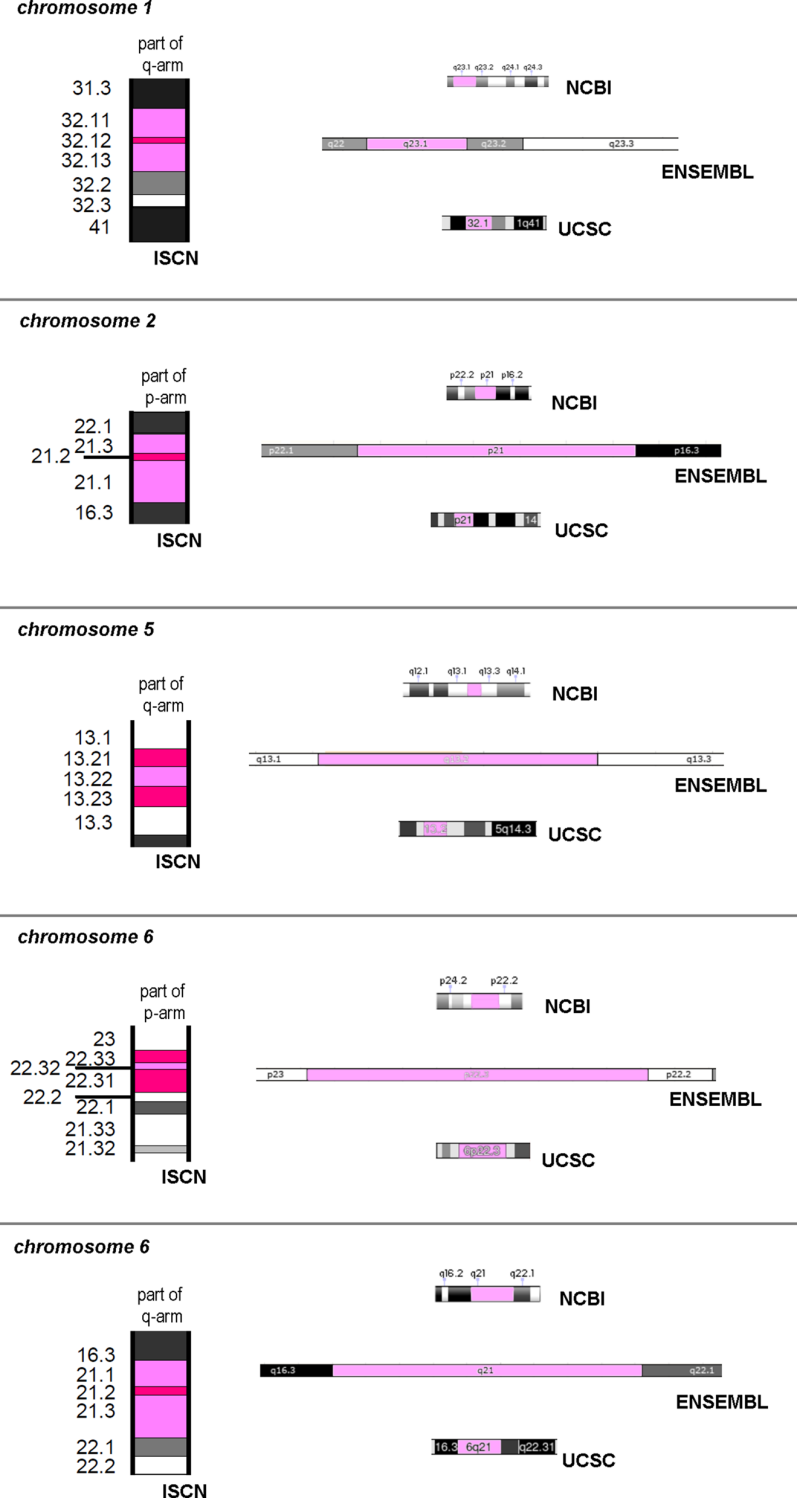
Here 15 subbands on chromosomes 1, 2, 5, and 6 are highlighted in pink, which are not included in genome browsers, but defined in ISCN

Obviously, the 850 band level of a haploid human chromosome set was originally used to align the GRC-sequencing data with chromosomal subbands. However, during this transfer, the splitting of 5 bands into three subbands each was missed. Thus, 15 subbands as shown in Fig. 2 are not included in all genomic browsers. There are depicted there as (i) 1q32.1 instead of 1q32.11, 1q32.12 and 1q32.13, (ii) 2p21 instead of 2p21.3, 2p21.2 and 2p21.1, (iii) 5q13.2 instead of 5q13.21, 5q13.22 and 5q13.23, (iv) 6p22.3 instead of 6p22.33, 6p22.32 and 6p22.31, and (v) 6q21 instead of 6q21.1, 6q21.2 and 6q21.3.

On the other hand, subband 9q34.1 is not subdivided in ISCN, but in GRC there are 3 subbands as 9q34.11, 9q34.12 and 9q34.13; the same was done for 6p24, which is divided in 6p24.3, 6p24.2 and 6p24.1. Interestingly, until version GRCH37/hg19 band 2q12.2 was also divided into 3 subbands (2q12.21, 2q12.22, 2q12.23) not present in ISCN, ever.

### Size of chromosomal subbands cannot be changed according to sequence results

**Fig. 3:**
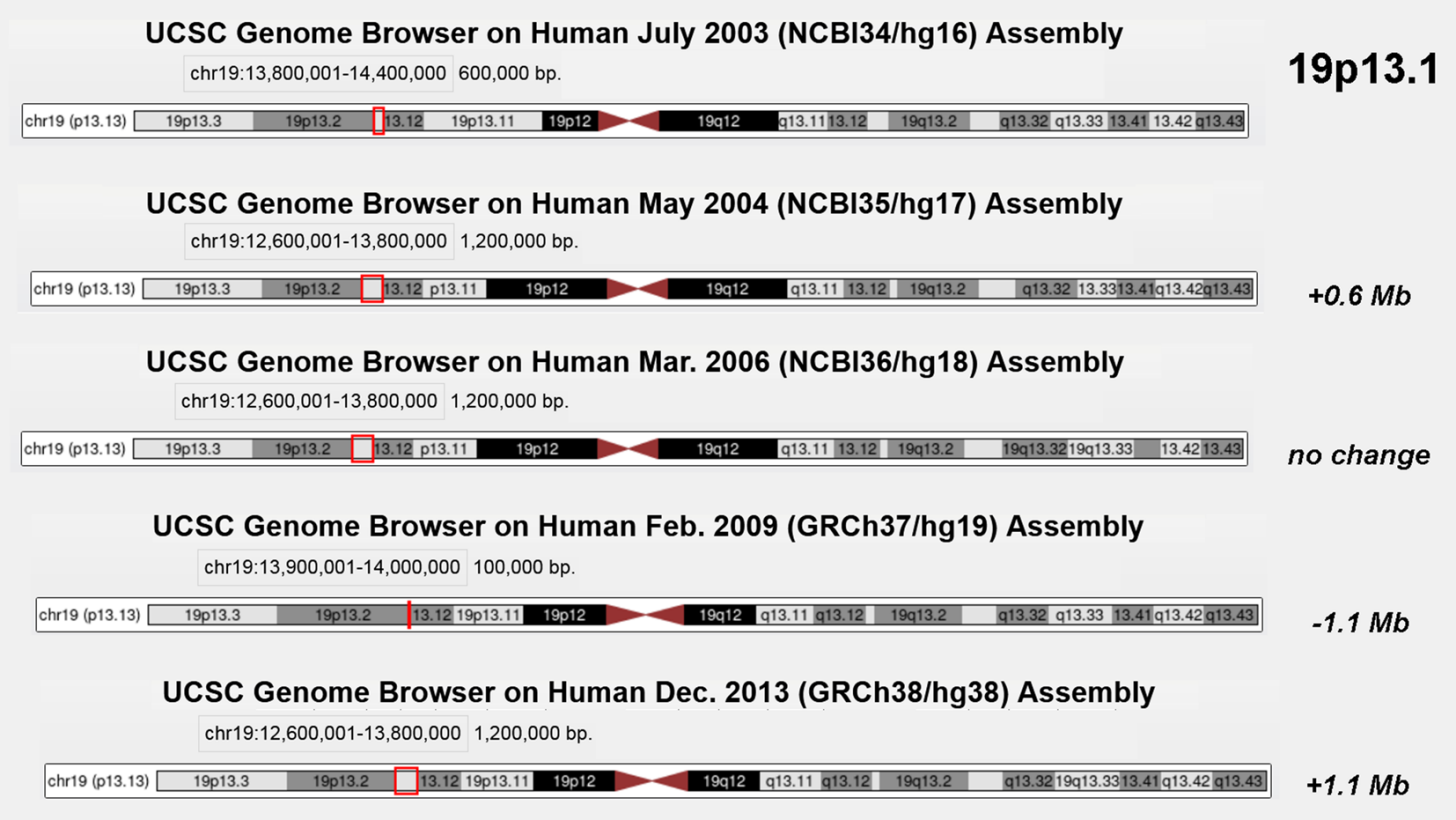
Change of band sizes in genomic browsers exemplified on subband 19p13.1 in five UCSC browser versions between 2003 and 2013

The sizes of the chromosomal subbands shown in ISCN were determined based on microscopic observations. Even though it is stated in ISCN [15] that “location and width of bands are not based on any measurements”, they represent what is and was seen by ten thousands of cytogeneticists worldwide, and since decades; thus it can be taken as fact, and no better data is available than this. Accordingly, individual band extensions in the chromosomal idiograms depicted in the genome browsers are not allowed to be changed based on results of sequencing. However, the latter has obviously been done when updating the browsers with new versions (Fig. 3). It could not be found out how GRC aligns sequencing data to GTG-bands—however, the described changes of chromosomal band sizes suggest that it has been done possibly the following way: In the first version several years ago it was defined according to the knowledge from that time that e.g. band A on a certain chromosome is located between DNA-markers X and Z. Later on more or less DNA-stretches had been found to be located indeed between these markers, and thus band size had to be adapted. It must be repeated—this is illegitimate and must lead to non-compatible molecular cytogenetic and molecular data (Fig. 3). The percentages / expansions of the chromosomal subbands can only be oriented on the values given for each band in Additional file 1: Table 1—column C. In Additional file 1: Table 1 size of chromosomal subbands were determined (in percent of total chromosomal length) according to ISCN (2020) [15] and aligned with the overall DNA-content per chromosome given in UCSC [9]. It was calculated:

$$x = \frac{{{\text{ A }}\left[ {{\text{Mb}}} \right]{\text{ x B }}\left[ {\text{\%}} \right]}}{100}$$

A = length of chromosome A [Mb]; B = percentage of a chromosomal band of the chromosome the band is located on.

### Chromosomal subbands cannot be seen in microscope if they are smaller than 1.0 megabasepair

According to an “American College of Medical Genetics guideline on the cytogenetic evaluation of the individual with developmental delay or mental retardation” statement from 2005, “at resolutions > 650 bands, alterations as small as 3–5 Mb can be reliably detected using chromosome analysis on peripheral blood; for the detection of subtle rearrangements in patients with either abnormal or normal karyotypes, molecular cytogenetic analysis may be useful” [19]. Also in high resolution GTG-banding a resolution of ~ 1400 bands can be achieved—this means that at this band level it may be (theoretically) possible to see bands of 1–2 Mb in size—maybe exceptionally, even 0.5 Mb. Deduced from that it may be optimistically suggested that at a resolution of 850 bands a subband may be seen if is at least 1 Mb in size. In Table 1 34 subbands are listed which are 0.1 to 0.9 Mb in size according to GRCh38/hg38. On the other hand, only 22 subbands are between 0.53 and 0.99 Mb according to ISCN and  Additional file 1: Table 1—column C (Table 2). None of them is smaller than 0.5 Mb and 2/3 of them are GTG-light bands; no GTG-dark band is smaller than 0.75 Mb. Here it is of interest, as above mentioned, that GTG-light bands are maximally decondensed—thus, higher banding resolutions than 1 Mb seem to be possible here.

**Table 1:** Chromosomal bands smaller than 1.0 Mb in size, according to UCSC Genome Browser (GRCh38/hg38) assembly are listed and compared to the size calculated based on ISCN (2020) idiograms (see Additional file 1: Table 1)

Chromosome	Bands smaller than 1.0 MB	Mb in UCSC	Mb acc. to ISCN	GTG banding color
#1	1p31.2	0.8	1.79	Dark
	1q42.11	0.5	2.15	Light
#2	2q23.2	0.6	1.90	Dark
	2q36.2	0.9	0.76	Light
#3	3p21.33	0.5	3.00	Light
	3p21.31	0.1	1.91	Light
	3p11.2	0.7	2.45	Light
	3q12.2	0.9	1.63	Dark
#5	5q12.2	0.3	1.96	Light
#6	6q11.2	0.1	2.13	Light
	6q22.2	0.2	2.66	Light
	6q23.1	0.9	3.72	Light
#7	7p15.1	0.9	7.77	Light
	7q22.2	0.7	2.78	Dark
#8	8q11.22	0.4	5.43	Dark
	8q12.2	0.7	2.00	Light
	8q21.12	0.1	2.29	Light
	9p11.1	0.8	2.19	Dark
#10	10p15.2	0.8	1.36	Dark
	10p12.32	0.1	0.82	Light
	10q24.33	0.9	1.09	Light
#11	11q22.2	0.7	1.63	Light
#12	12q24.12	0.6	1.66	Dark
#13	13q14.12	0.6	1.69	Dark
#14	14q32.32	0.8	2.18	Dark
#17	17q21.1	0.4	3.23	Light
	17q23.1	0.7	2.98	Light
	17q25.2	0.4	0.99	Dark
#19	19q13.13	0.4	4.29	Light
#20	20q13.11	0.4	1.94	Light
Y	Yp11.32	0.3	4.09	Light
	Yp11.31	0.3	3.71	Dark
	Yp11.1	0.1	2.23	Dark
	Yq11.1	0.2	2.23	Dark
Overall	34 subbands	17.8	84.64	14 Dark 20 Light

Also it is indicated of the corresponding band was GTG-dark or GTG-light

**Table 2:** Chromosomal bands smaller than 1.0 Mb in size (size calculated based on ISCN (2020) idiograms—see Additional file 1: Table 1) compared to their size according to UCSC Genome Browser (GRCh38/hg38) assembly are listed. Also it is indicated of the corresponding band was GTG-dark or GTG-light

Chromosome	GTG banding color	Bands smaller than 1.0 MB	Mb acc. to ISCN	Mb in UCSC
#1	Light	1p36.33	0.72	2.3
#2	Dark	2p21.2	0.76	2.0
	Light	2q36.2	0.76	0.9
#3	Light	3p26.2	0.82	1.2
	Light	3q13.32	0.65	1.7
#5	Light	5p14.2	0.84	1.3
#6	Light	6p22.32	0.80	3.4
	Dark	6p21.32	0.80	1.4
	Light	6p11.2	0.53	1.3
#7	Light	7p21.2	0.83	2.8
	Light	7q21.12	0.56	1.8
#10	Light	10p12.32	0.82	0.1
	Light	10q23.32	0.82	1.2
#12	Light	12q21.32	0.55	2.3
#13	Light	13q33.2	0.84	2.2
#14	Light	14q31.2	0.82	1.3
#16	Dark	16q22.2	0.79	2.0
	Dark	16q24.2	0.79	1.7
#17	Dark	17q24.1	0.75	1.6
	Dark	17q25.2	0.99	0.4
#22	Dark	22q11.22	0.99	1.4
X	Light	Xp11.21	0.77	3.3
overall	7 dark 15 light	22 subbands	17.00	37.6

Overall this means, band length in GRC should be reconsidered, not allowing smaller bands in size than 0.5 Mb; especially as such are even in UCSC no more visible (Fig. 3). Furthermore, it should be considered GTG-light bands contain naturally less DNA than GTG-dark bands. A simple projection of the same stretch of DNA in a GTG-light band as in a GTG-dark band will not reflect what is biological reality.

## Lessons to learn from classical molecular genetics and molecular cytogenetics

### What about repetitive DNA?

In early times of molecular genetics (here referred to as “classical molecular genetics”) the interest in repetitive DNA within the human genome was immense. It was simply accessible and easy to study. As summarized elsewhere [20], those studies produced immense data about these yet by modern approaches (like second generation sequencing) still almost not accessible regions of the human genome [5]. In Table 3 just a selection of since decades identified satellite DNA-sequences is given. Most of them were and are used in millions of FISH experiments (here referred to as “molecular cytogenetics”) and the location of these DNA-stretches is more than proven. Even the approximate sizes of these repetitive DNAs are known. If only those satellite DNAs from Table 3 would be included into the genome browsers, in one run 100 Mb of yet not mapped DNA-regions could be filled. This is also more than timely, as the expression of such satellite DNAs has been shown as least as long non-coding RNAs not only just recently [21]. Additional megabases could be filled by using the information in parts collected elsewhere [20] and also by adding nucleolus organizing regions to all acrocentric p-arms and telomeric sequences to the chromosomal ends. From the view of a biologist it is somehow surprising to start and end each chromosome in the browsers ignoring there the well-known telomeric repeats—they should be included, as they cannot be searched in UCSC; NCBI or Ensembl, yet. Maybe, database of genomic variants [22] might start thinking about inclusion of polymorphisms in repetitive DNAs, too. Finally and interestingly, without taking into account these megabases of repetitive DNA GRC includes nonetheless overall 3,091,153,988 base pairs in the human sequence (Additional file 1: Table 1); an incredible exact number, considering all the yet unknown regions.

**Table 3:** Satellite DNAs with known location and sizes according to [14]

Chromosome band	name	Size (Mb)	Average size (Mb)	Satellite
1p11.1q11	D1Z7	1.90	1.90	Alpha
5p11q11.1	D5Z2	2.00–4.00	3.00	
19q11	D19Z3	2.00	2.00	
1q11q12	D1Z5	0.44–1.90	1.17	Alpha
1q12	D1Z1	n.a	n.a	III
2p11.1q11.1	D2Z1	1.05–2.90	2.00	Alpha
3p11q11.1	D3Z1	2.90–3.40	3.15	Alpha
3p11q11.1	VIIB4	2.75	2.75	Alpha
4p11q11	D4Z1	3.20–5.00	4.10	Alpha
4p11q11	p4n1/4 and pG-Xba11/340	0.40	0.40	Alpha
9p11.1q11		0.400.40	0.40	
5q11.1	D5Z1	0.04–0.25	0.15	Alpha
6p11.1q11.1	D6Z1	3.00	3.00	Alpha
7p11.1	D7Z2	0.01–5.50	2.25	Alpha
7q11.1	D7Z1	1.60–3.81	2.70	Alpha
8p11.1q11.1	D8Z2	1.00–2.55	1.80	Alpha
9p11.1	D9Z5	1.25	1.25	ß
9q12		1.25	1.25	
9p11.1q11	D9Z4	2.70	2.70	Alpha
9q12	D9Z3	n.a	n.a	III
10p11.1	n.a	0.08–0.53	0.30	II
10p11.1q11.1	D10Z1	1.02–2.02	1.52	Alpha
10q11.1	n.a	0.03–0.62	0.32	II
11p11.1q11	D11Z1	0.85–4.76	2.80	Alpha
12p11.1q11	D12Z3	1.40–4.30	2.90	Alpha
13p11.1q11	D13Z1	1.80–2.30	2.00	Alpha
21p11.1q11.1	D21Z1	0.40–3.15	1.78	
14p11.1q11.1	D14Z1	1.36–2.30	1.83	Alpha
22p11.1q11.1	D22Z1	2,00–2.60	2.30	
15p11.2	D15Z1	2.50–3.00	2.75	III
15p11.1	D15Z4	2.50–4.50	3.50	Alpha
15q11.1	D15Z3	2.50–4.50	3.50	Alpha
16p11.1q11.1	D16Z2	1.40–2.00	1.70	Alpha
16q11.2	D16Z3	4.00	4.00	II
17p11.1	D17Z1-B	0.50–0.90	0.70	Alpha
17p11.1q11.1	D17Z1	1.00–2.70	1.90	Alpha
18p11.1	D18Z2	1.70	1.70	Alpha
18p11.1q11.1	D18Z1	1.36	1.36	Alpha
19p11	D19Z2	n.a	n.a	Alpha
20p11.1	D20Z2	1.02	1.02	Alpha
20q11.1	D20Z1	3.90	3.90	Alpha
22p11.2p11.1	D22Z4	n.a	n.a	Alpha
22q11.1	D22Z21	0.12–2.80	1.50	Alpha
Xp11.1q11.1	DXZ1	1.38–3.73	3.25	Alpha
Yp11.1q11.1	DYZ3	0.29–1.02	0.65	Alpha
Yq12	DYZ1	6.80–13.60	10.40	II
Yq12	DYZ2	4.20	4.20	III
Yq12	GAATG	0.30–10.00	5.15	II
**Overall**			** ~ 100**	

## Conclusion

GRC and human genome browsers would tremendously profit in their comprehensiveness and accuracy if ‘classical (cyto)genetic’ data and ‘karyotype coding’ [12] would be considered more. As nicely stated by Iourov, Yurov and Vorsanova in 2020 [23]: “Undoubtedly, genome-centric and gene-centric are the words to describe actual concepts in human genetics. In a world of genes and genomes, the lack of required attention to chromosomes is often observed. As a result, chromosome research gradually loses the genetic (genomic) context. Certainly, brilliant insights into chromosome biology obtained by studies dedicated to molecular/cell biology, evolution, biochemistry, biophysics, etc., are fascinating. However, genome research and human (medical) genetics miss the essential link between genes and genomes, which is determined by chromosomal analysis (i.e., cytogenetics, molecular cytogenetics, cytogenomics). This is also the case for diagnostic research, which has recently suffered problems in quality of cytogenetic diagnosis. Data on genes and genomes are useless outside the chromosomal context when intrinsic molecular and cellular pathways are highlighted in health and disease. Without the chromosomal context, genes are virtual elements interacting with each other in an elusive digital universe. Unfortunately, this situation is generally the case for numerous attempts to analyze and interpret genomic data. More dramatically, education programs in genomics and genomic medicine developed for medical/biological students, physicians, or the public generally conceal any information about the chromosome, the physical (biological) storage of genomic data” [23]. This statement is further underlined by publicationos of Ron Hochestenbach and colleagues [24, 25].

Yet, and also after inclusion of more data in future, the results shown in the browsers are nothing more than a model of the human genome—they do not depict the natural human genome, they do not describe the highly variable nature within living cells which is present in a three dimensional context. GRC, as ISCN provide both mainly a unifying nomenclature, to be able to describe aberrations from the norm.

## Supplementary Information

**Additional file 1.** Here sizes of chromosomal subbands were determined (in percent of total chromosomal length) according to ISCN (2020) [15] and aligned with the overall DNA-content per chromosome given in UCSC [9].

## Data Availability

All data generated or analysed during this study are included in this published article and its supplementary information files.

## Abbreviations

GRC: Genome Reference Consortium
GTG: G-bands by trypsin using Giemsa
HGP: Human Genome Project
ISCN: International System for Human Cytogenetic or Cytogenomic Nomenclature
NCBI: National Center for Biotechnology Information
TADs: Topologically associating domains
UCSC: University of California, Santa Cruz
